# Genetic, morphometric, and molecular analyses of interspecies differences in head shape and hybrid developmental defects in the wasp genus *Nasonia*

**DOI:** 10.1093/g3journal/jkab313

**Published:** 2021-09-02

**Authors:** Lorna B Cohen, Rachel Jewell, Dyese Moody, Deanna Arsala, John H Werren, Jeremy A Lynch

**Affiliations:** 1 Biological Sciences, University of Illinois at Chicago, Chicago, IL 60607, USA; 2 Optical Imaging Core, Van Andel Institute, Grand Rapids, MI 49503, USA; 3 Department of Biology, University of Rochester, Rochester, NY 14627, USA; 4 Department of Ecology and Evolution, University of Chicago, Chicago, IL 60637, USA

**Keywords:** epistasis, complex traits, *Nasonia*, hybrid compatibility, morphological development

## Abstract

Males in the parasitoid wasp genus *Nasonia* have distinct, species-specific, head shapes. The availability of fertile hybrids among the species, along with obligate haploidy of males, facilitates analysis of complex gene interactions in development and evolution. Previous analyses showed that both the divergence in head shape between *Nasonia vitripennis and Nasonia giraulti*, and the head-specific developmental defects of F2 haploid hybrid males, are governed by multiple changes in networks of interacting genes. Here, we extend our understanding of the gene interactions that affect morphogenesis in male heads. Use of artificial diploid male hybrids shows that alleles mediating developmental defects are recessive, while there are diverse dominance relationships among other head shape traits. At the molecular level, the sex determination locus *doublesex* plays a major role in male head shape differences, but it is not the only important factor. Introgression of a *giraulti* region on chromsome 2 reveals a recessive locus that causes completely penetrant head clefting in both males and females in a *vitripennis* background. Finally, a third species (*N. longicornis*) was used to investigate the timing of genetic changes related to head morphology, revealing that most changes causing defects arose after the divergence of *N. vitripennis* from the other species, but prior to the divergence of *N. giraulti* and *N. longicornis* from each other. Our results demonstrate that developmental gene networks can be dissected using interspecies crosses in *Nasonia*, and set the stage for future fine-scale genetic dissection of both head shape and hybrid developmental defects.

## Introduction

Development in multicellular organisms involves complex interactions of differentiating tissues and cells. These cell and tissue level interactions are, in turn, governed by interactions among genes that are part of gene regulatory networks (GRNs) (Davidson *et al.* 2003; Peter and Davidson 2011). Differences in shape within populations and between species are presumably encoded by changes in the identity or magnitude of connections within and between developmental GRNs (Stathopoulos and Levine 2005; Hinman and Davidson 2007). However, identification of the molecular basis of shape differences is rare.

Quantitative trait locus (QTL) analyses have typically found numerous heritable loci participating in regulating the shape of even relatively simple structures (Klingenberg *et al.* 2001; Mezey *et al.* 2005). The magnitude of the effects of each locus on shape may be individually small, making isolating and identifying the causative genes that much more difficult. In addition, complex, nonadditive (termed collectively “epistatic” here) interactions among loci are also known to be important, and the effects of epistatically interacting alleles will often be missed in typical QTL analyses, due to recessivity of some interactions, and are difficult to detect even when they are specifically sought (Carlborg and Haley 2004; Laurie *et al.* 2014).

Knowledge of the causes and consequences of epistatic interactions within developmental GRNs will deepen our understanding of how complex traits are encoded in the genome, how epistatic interactions affect shape and size, and how developmental GRNs evolve (Phillips 2008; Mackay 2014). Because candidate genes mediating complex epistasis are usually not obvious, a forward genetic approach is appropriate to identify participating loci. However, this approach has its own drawbacks, as intraspecies trait differences can be too subtle to reliably identify causative loci. In addition, interspecific models that can both make fertile hybrids, and also have strong morphological differences, are rare.

We, and others, have been developing parasitoid wasps in the genus *Nasonia* as a model system that is well suited for investigating evolutionary genetics (Beukeboom and Desplan 2003; Werren and Loehlin 2009; Lynch 2015). There are four species in the genus that can all be crossed to produce viable and fertile hybrids. In addition, *Nasonia* are haplodiploid, meaning that unfertilized eggs produce haploid males, and fertilized eggs become diploid females (Werren and Loehlin 2009; Lynch 2015). Combining these two features provides a major advantage of the system for evolutionary genetics. Viable F1 inter-species hybrid females can be set as virgins, and they will produce large broods of haploid, recombinant F2 males. Not only are recessive traits exposed in the hemizygous genome of these males, but so are more complex interactions that typically require several rounds of crossing to produce complex homozygotes in traditional diploid systems (Werren and Loehlin 2009; Lynch 2015). Aside from forward genetic approaches described above, *Nasonia* is amenable to reverse genetic approaches, such as RNA interference (Lynch and Desplan 2006; Werren *et al.* 2009). The utility of the *Nasonia* genus model system to identify the genetic basis of a wide variety of biological traits of evolutionary importance has been amply demonstrated in recent years (Gadau *et al.* 2002; Verhulst *et al.* 2010; Loehlin and Werren 2012; Brucker and Bordenstein 2013; Niehuis *et al.* 2013; Hoedjes *et al.* 2014; Martinson *et al.* 2017; Funkhouser-Jones *et al.* 2018; Pannebakker *et al.* 2020; Zou *et al.* 2020).

The morphological differences among *Nasonia* species are primarily features of haploid males, helping to make genetic analysis of the evolution of shape in this species tractable. There are striking differences in head shape between males of the species *Nasonia vitripennis* and *Nasonia giraulti* (Darling and Werren 1990). Our subsequent QTL analyses showed that these differences were strongly affected by interactions among several loci. We also observed that complex epistatic interactions give rise to developmental defects in a large proportion of F2 hybrid males (Werren *et al.* 2016). The most prominent among these defects were facial midline clefting, and asymmetries between the left and right sides of the face (Werren *et al.* 2016). We believe that these hybrid defects are also important to understand, as they represent the potential for negative allelic interactions to constrain morphological evolution, which may limit the paths evolution can take in response to selection.

Here, we aim to better understand the genetic and evolutionary basis of head shape differences between species, and how they relate to the defects in the hybrid males using the powerful genetic tools available in *Nasonia*. An important addition in this analysis relative to our previous work is the inclusion of a third species, *N. longicornis*, which is a close relative of *N. giraulti* (separated by ∼400,000 years, while both *N. longicornis and N. giraulti* diverged from *N. vitripennis* about 1.4 million years ago (MYA) (Raychoudhury *et al.* 2010; Martinson *et al.* 2017)). We used crosses among these three *Nasonia* species to investigate the evolutionary history of alleles mediating hybrid incompatibility. We also investigated the role of the conserved sex differentiation factor *doublesex* in generating species and sex-specific head shape features among *Nasonia* species. Experimentally generated diploid males were used to investigate dominance relationships of alleles at loci affecting head shape, and showed that alleles mediating developmental defects are recessive. Finally, we characterized an introgression of a genomic interval from *N. giraulti* into an *N. vitripennis* background to demonstrate the separability of alleles involved in hybrid incompatibilities affecting head shape abnormalities. Overall our results show that the combination of forward evolutionary genetics, reverse genetics with candidate genes, and morphometric analyses make *Nasonia* head shape a useful model system for studies of evolutionary developmental biology.

## Materials and methods

### Hybrid crosses

Highly inbred, and *Wolbachia* free strains of *N. vitripennis* (AsymCx), *N. giraulti*, (RV2x), and *N. longicornis* (IV7) (Werren *et al.* 2010) were used to make hybrids (*Wolbachia* infections normally prevent hybrid production). A fourth species, *N. oneida*, was not used in this study (Raychoudhury *et al.* 2010). For each cross, a ratio of 15 females to nine males were allowed 24 h to mate before females were provided fly (*Sarcophaga bullata*) hosts to parasitize. Fifteen to twenty F1 hybrid virgin females from each interspecies cross were then provided hosts to parasitize. Setting females as virgins guarantees all offspring to be haploid males.

### Measurements

For all species, hybrids, and RNAi affected wasps heads were stained, mounted, imaged, and measured as described previously (Werren *et al.* 2016). Acronyms are as follows: MHW, maximum head width; HL, head length (HL); OIO, interocular distance through ocelli; MIO, maximum interocular distance; AIO, interocular distance across antennal sockets; FE, distance from bottom of eye to center of mandible; FEP, farthest point on cheek perpendicular to line FE (see Supplementary Figure S1 for diagram). Measurements are presented as ratios to normalize natural difference in overall size of the individual. MHW, OIO, MIO, and AIO are normalized in relation to HL and dividing FEP by FE normalizes cheek size. We refer to these normalized values throughout the text. Mann–Whitney *U*-tests were performed for nonparametric data between two groups, and Bonferroni adjustments were made for multiple comparisons. For wild-type parent species, comparisons were made among males of each species, among females of each species, and between males and females within each species. Each experimental group was compared individually to *N. vitripennis* and *N. giraulti* wild-type males. Plots were generated using R (R-Core-Team 2017), raw averages, standard deviations, and significance can be found in Supplementary Tables S1 andS3.

### Analyses of symmetry

#### Heads

Head symmetry was measured by Procrustes distance analysis (Rohlf and Slice 1990; Goodall 1991) of 105 hybrid male heads as well as 58 wild types (30 *N. vitripennis* and 28 *N. giraulti*, split evenly between males and females). Each head was marked at 16 landmarks: One at each ocellus, at the tops and bottoms of each eye, at the maximum arc of each eye, the maximum arc of each cheek, the center point of the mandible, both ends of the MIO, and location of each antennal socket (Supplementary Figure S1). Landmarks were established three times for each head and coordinates for each landmark were averaged and imported as an array in R (R-Core-Team 2017)Scaling, rotating, and superimposition of head landmarks was carried out using R packages gemorph, shapes, and Momocs (Bonhomme *et al.* 2014; Adams *et al.* 2017; Dryden 2019). R package vegan (Oksanen *et al.* 2017) quantifies symmetry by overlaying the left and right sides of heads and performs Procrustes distance analyses, defined as **Σ**[(distance between corresponding landmarks)^2^].

#### Legs and wings

Front wings and T1 legs of the same 105 hybrid and 72 wild-type wasps were carefully removed and mounted on slides. Each wing and leg were imaged on a Zeiss Stereo Discovery V.8 dissecting scope using Zeiss Axiovision software v. 4.8. Each specimen was measured three times and the length averaged. The difference in length between left and right sides of each appendage was compared for hybrids and wild types.

### RNAi

#### Diploid male production

To generate diploid males, we used parental RNAi (Lynch and Desplan 2006) on a mutant strain of *N. vitripennis* with grey eye color (*N.vit^Oy^*^/Oy^). Female yellow pupae of *N.vit^Oy^*^/Oy^ were injected with 1ug/ul of double-stranded RNA (dsRNA) targeting *Nv-transformer (Nv-tra)*, whose function is required for female development in fertilized eggs (Verhulst *et al.* 2010). The injected *N.vit^Oy^*^/Oy^ adult females were then crossed to the wild type *N. giraulti* (RV2x), which have a red-brown eye color. While haploid males display the grey eye phenotype, the hybrid, diploid males express wild type red-brown eye color allele obtained from the *N. giraulti* parent. Male *vs* female offspring are easily differentiated in the pupal state by wing size and absence/presence of an ovipositor (Werren and Loehlin 2009).

Primer Sequences (Arsala and Lynch 2017):

*Nv*-transformer-F: ggccgcgggcaaaatccgtgagacaac

*Nv*-transformer-R: cccggggcgaggctgtcggcaaaaata

#### *Ng-dsx* knockdown

Knockdown of *N. giraulti doublesex (Ng-dsx)* was carried out by injecting *N. giraulti* larvae with dsRNA (Werren *et al.* 2009) targeted to *Ng-dsx*. Mid-stage larvae collected ∼8 days after egg laying were positioned on double-sided tape on a slide for injection. Larvae were returned to 25**°** incubator to eclosion. Adult heads were stained, imaged, and measured as described above.

*Ng*-doublesex-F: ggccgcggcgcggaaagttgaagaagtc

*Ng*-doublesex-R: cccggggcaatccaagtcccacatctgc

### Introgressions

Introgression of *Ng* chromosomal regions into an *Nv* genetic background is routinely used to investigate the genetic basis of differences in traits between *Nasonia* species, and some cases for positional cloning of causal loci. In a previous study, a region on chromosome (Chr) 2 was implicated in abnormal head clefting in F2 males (Werren *et al.* 2016). We had generated an introgression of this region from *N. giraulti* into *N. vitripennis* to examine its role in head morphology without interference from other loci that would be co-inherited in F2 haploid males*.* The initial Chr 2 introgression line is designated INT_2C, and head shape effects were observed, in addition to phenotypic effects on body color, survival, and female fertility (data not shown). Subsequent recombinants were generated by using primers flanking insertion/deletion differences across the region. A smaller-scale introgression designated 2C-Cli was produced that shows an abnormal head clefting in both males and females. The recessive lethal and female fertility effects were separated from the clefting region by recombination. Both introgression lines were generated according to previously described methods (Breeuwer and Werren 1995). The smaller region is estimated to be 16 centimorgan based on the *Nasonia* fine-scale map (Desjardins *et al.* 2013).

A line with an introgression on Chr 4 (denoted INT_wm114) had previously been generated to study the sex-specific wing size differences in *Nasonia* (Loehlin *et al.* 2010), and contains the sex determination locus *doublesex* (*dsx*) from *N. giraulti* in a *N. vitripennis* genetic background. We utilized this strain to further examine the role of *dsx* in head shape differences between the sexes and among the species. Adult heads were stained, imaged, and measured as described above.

## Results

### Large differences among male and subtle but significant differences among female head shape in the *Nasonia* genus

Building on previous work (Darling and Werren 1990; Werren *et al.* 2016), we produced a set of normalized measurements of the heads of males and females from *N. vitirpennis (*Nv), *N. giraulti* (Ng), and *N. longincornis* (Nl) (see *Materials and Methods*, Supplementary Figure S1). This allows comparison of the wild-type head shapes as well as head shapes resulting from experimental manipulation. In general, the heads of females are very similar among the species. However, we were able to detect some subtle, yet significant, differences. For example, normalized maximum head width (MHW/HL) of Nv is significantly wider than for the other species (Figure 2A). In addition, the normalized cheek size (FEP/FE) of Nv females is significantly smaller than both Ng and Nl females. Finally, the normalized interocular width of Nv female heads is larger than that of Ng females, but not Nl females.

**Figure 1:**
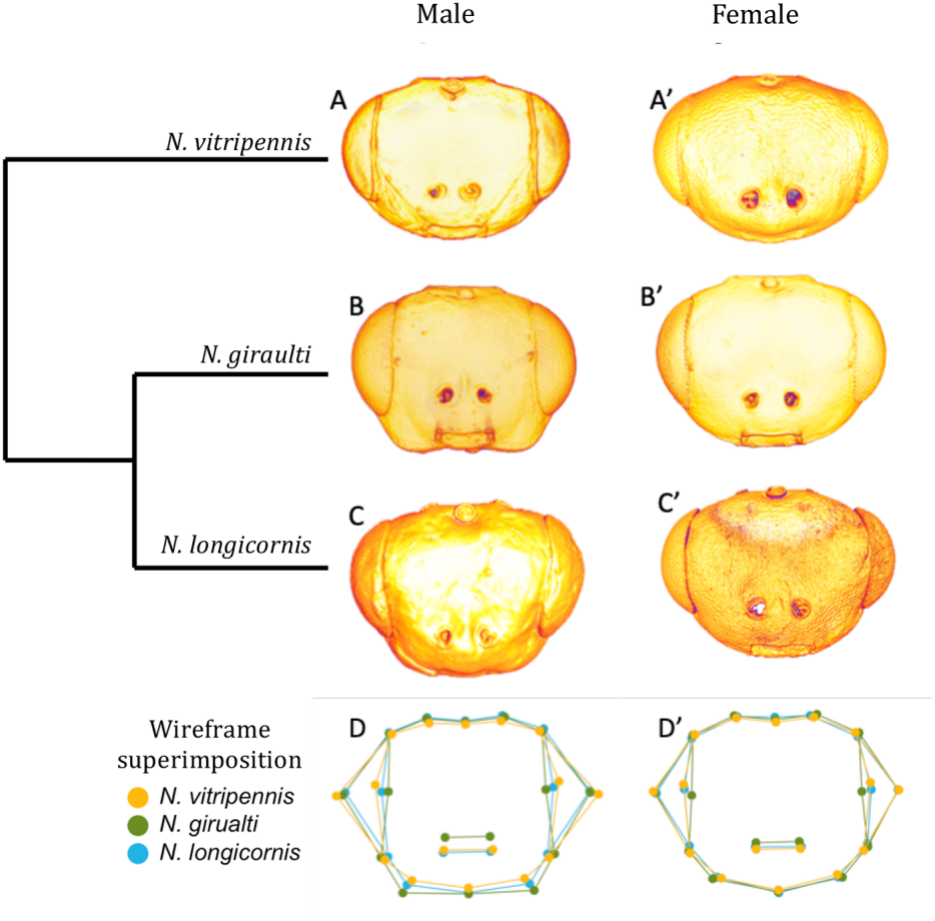
Shape differences among wild type species. (A–C’) Representative images of wasp heads. (D–D’) Procrustes superimposition of average wild type head shapes based on 16 landmarks. Morphology recapitulated by wireframe diagram. (A) *N. vitripennis* male, (A’) *N. vitripennis* female, (B) *N. giraulti* male, (B’) *N. giraulti* female, (C) *N. longicornis* male, (C’) *N. longicornis* female, (D) Superimposed wireframe diagrams of male heads (D’) Superimposed wireframe diagrams of female heads. Yellow landmarks denote *N. vitripennis*, green *N. giraulti*, and blue *N. longicornis.*

**Figure 2:**
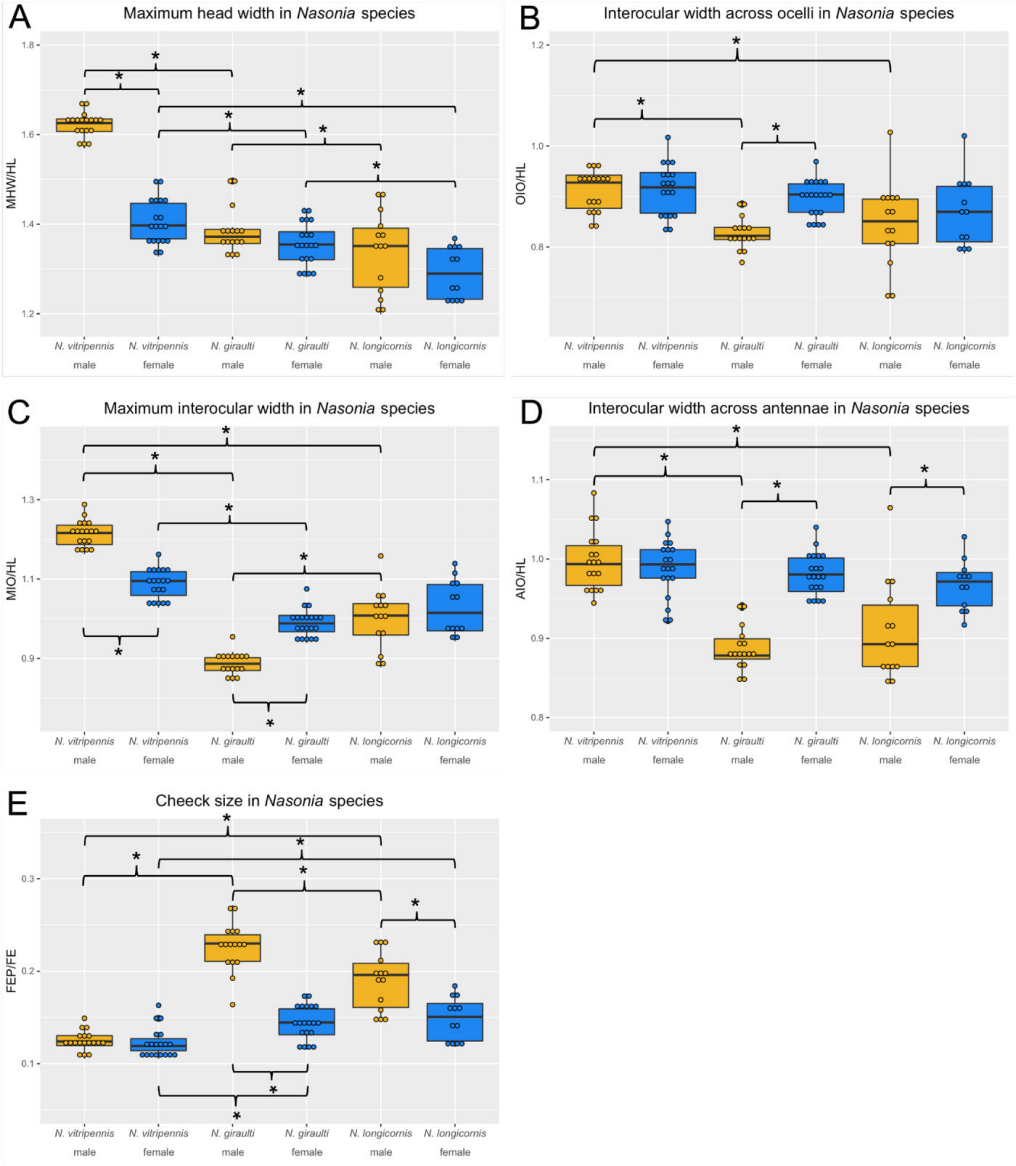
Measurement ratios of each parent species presented as box and whisker plots. Each dot represents a single individual, a box represents the inter-quartile range, the center line represents the median value and vertical lines represent upper and lower quartile ranges. (A) Maximum head width over head length (MHW/HL), (B) Interocular width at ocelli over head length (OIO/HL), (C) Maximum interocular width over head length (MIO/HL), (D) Interocular width at antennae over head length (AIO/HL), (E) Cheek size (FEP/FE.) Males are shown in yellow and females in blue. Comparisons were made among males of each species, among females of each species, and between males and females within each species. Asterisks indicate *P* < 0.05.

In contrast, large differences among the species occur in male head morphology, and the magnitude of sex-specific head shape differs between species. For example, male and female heads of Nv are similar for most measures (Figure 1, A and A’), except that the male heads have much larger normalized maximum head width (MHW/HL) and maximum interocular distance (MIO/HL) (Figure 2, A and C), giving them an exaggerated oval shape relative to Nv females. In contrast, Ng males are significantly diverged from Ng females by several measures. All of the normalized interocular width measurements [interocular distance through ocelli (OIO/HL), maximum interocular distance (MIO/HL), and interocular distance across antennal sockets (AIO/HL)] of the male Ng heads are significantly smaller than those of Ng females, and even more so than for Nv male heads (Figure 2, B–D). Ng males also have much larger normalized cheek size than Ng females and Nv males (Figure 2E), and it is speculated that this is due to larger mandibular gland underneath the exoskeleton. Overall these exaggerated dimorphic features give Ng males a distinctive square, jowly appearance, relative to the smooth elongated oval features of Nv males.

Nl males are similar to Ng males in all head shape characteristics, except that the divergence from conspecific females and Nv males are not as extreme as in Ng males. The means for all of the normalized interocular measurements are smaller for Nl males relative to Nl females, but the differences are smaller than those between the Ng sexes and are only statistically significant for the width at the antennae (Figure 2, B–D). Nl male cheeks are statistically significantly larger (Figure 2E) than Nl females', but again the magnitude of the difference is much smaller in comparison to the difference between the sexes in Ng (Figure 2E).

### The sex determination effector *doublesex* plays an important role in generating divergent head shape in *Nasonia* males

Since the divergent features of head morphology in *Nasonia* species are more pronounced among males, we hypothesized that the sex determination system plays an important role in generating the sex-specific divergences in head morphology. As male *N. giraulti* heads showed the most divergence from their conspecific females, and also from *N. vitripennis* males, we focused on the role of *doublesex in N. giraulti* head shape. The sex determination gene *doublesex* (*dsx*) is a major effector of primary sex determination pathway throughout metazoa, and it is also known to play a role in sex-specific somatic differences in developmental traits that vary between spieces (Hediger *et al.* 2004; Loehlin *et al.* 2010; Verhulst *et al.* 2010; Tanaka *et al.* 2011; Ito *et al.* 2013). The *d*sx locus has been characterized in *Nasonia* (Oliveira *et al.* 2009), and affects sex dependent, interspecific differences in wing size between *Nv* and *Ng* (Loehlin *et al.* 2010). Recently, knockdown of *Nv-dsx* by RNAi revealed that this gene is also important for male specific antenna pigmentation and in males (Wang *et al.* 2020). To examine the potential role of *dsx* orthologs in generating sex-specific head fates, we focused on Ng, where male head traits are most divergent from both its conspecific females and from Nv.

**Figure 3:**
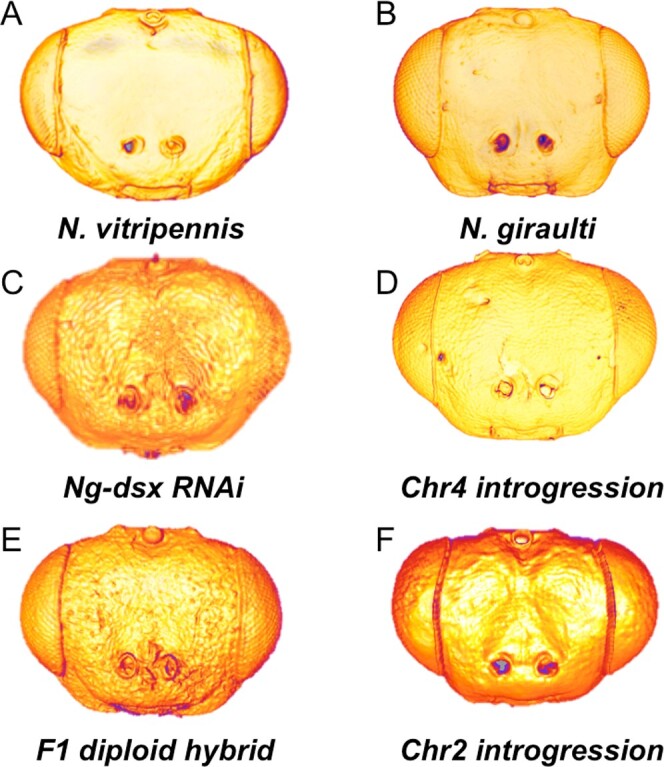
Experimental hybrid head shapes. (A) Wild type *N. vitripennis* male (B) Wild *type N. giraulti* male, (C) Diploid male, (D) *N.g. dsx* knockdown, (E) Introgression on Chr 2, (F) Introgression on Chr 4, arrowhead points to midline cleft. Note no other obvious asymmetries or abnormalities.

**Figure 4:**
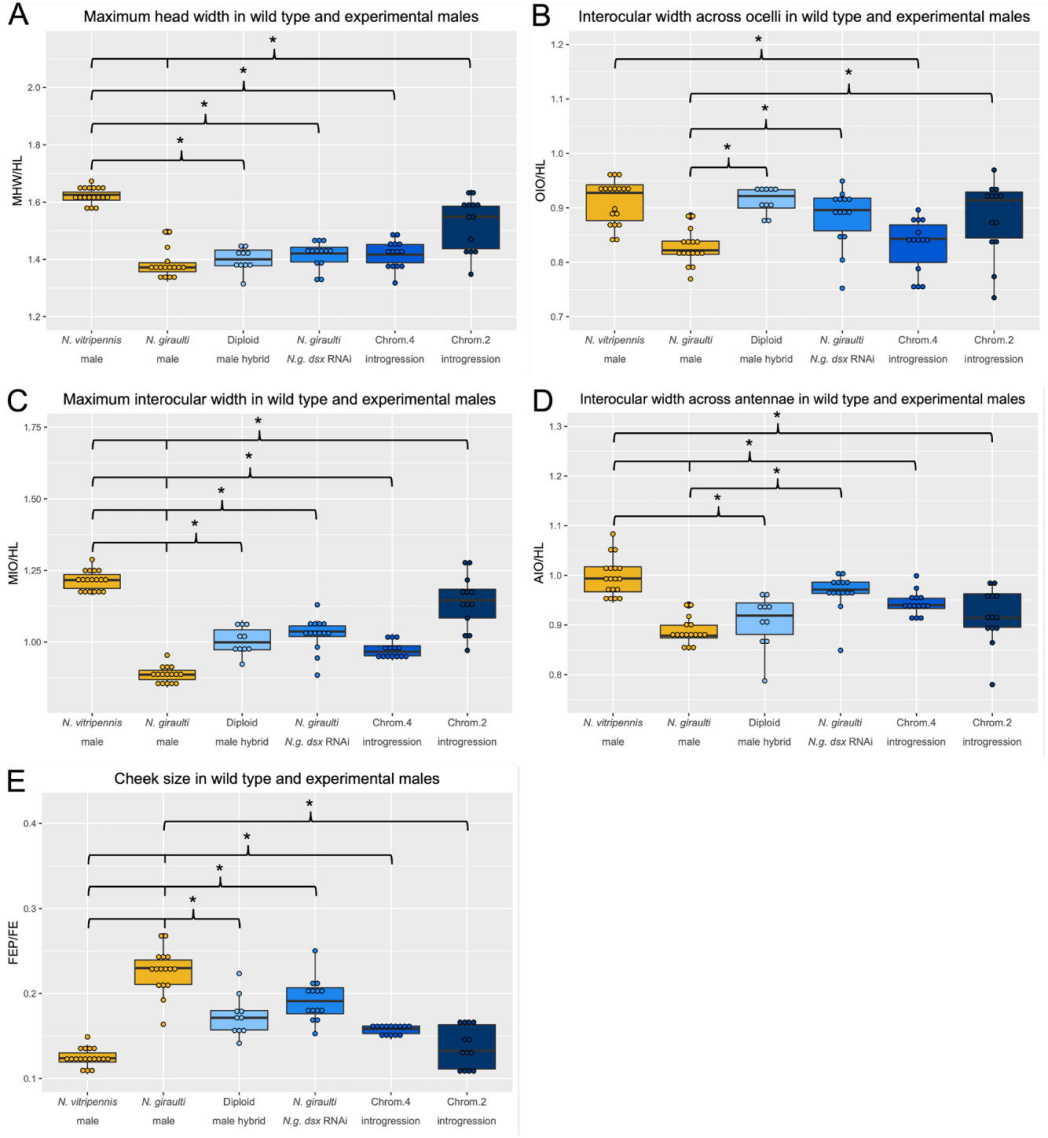
Measurement ratios of RNAi and introgression experiments, presented as box and whisker plots. Each dot represents a single individual, a box represents the inter-quartile range, the center line represents the median value and vertical lines represent upper and lower quartile ranges. (A) Maximum head width over head length (MHW/HL), (B) Interocular width at ocelli over head length (OIO/HL), (C) Maximum interocular widther over head length (MIO/HL), (D) Interocular width at antennae over head length (AIO/HL), (E) Cheek size (FEP/FE). Wild type *N. vitripennis and N. giraulti* males are shown in yellow and experimental lines in varying shades of blue. Each experimental group was compared to both wild type groups. Asterisks indicate *P* < 0.05.

Larval RNAi (Werren *et al.* 2009) was used to knock down *N. giraulti doublesex* (*Ng-dsx*) in male (progeny of virgin females) late-stage larvae before the main period of growth and patterning of the eye and antennal imaginal discs commenced. The distinctive features of Ng male heads were significantly altered by *Ng-dsx* knockdown (Figures 3 and 4, Supplementary Table S3). All of the normalized interocular width measures, which are narrower in Ng males relative to Ng females (and Nv males), are significantly larger, relative to wild-type Ng males, after *Ng-dsx* RNAi (Figure 4, B–D). In addition, *Ng-dsx* RNAi leads to significant reduction in normalized male cheek size (FEP/FE) relative to wild-type Ng males (Figure 4E).

From these results, we can conclude that *Ng-dsx* plays an important role in producing the male-specific head shape found in *Ng*, and that knockdown of *Ng-dsx* results in feminized head shape. This might suggest that the female form is the default, and that *Ng-dsx* acts only to masculinze the male head in Ng. However, the *Ng*-*dsx* RNAi males were still significantly different from *Ng* females at MHW/HL, MIO/HL, and normalized cheek size (Supplementary Table S2), indicating there was not a complete transformation to the female phenotype. This may indicate that either residual *Ng-dsx* (due to incomplete knockdown) was sufficient to partially produce male traits, or that additional factors not under the influence of *dsx* contribute to male head patterning in Ng. Further systematic testing of *dsx* orthologs among the sexes and species of *Nasonia* will be required to distinguish these possibilities, but these are beyond the scope of this study.

### Introgression of *a Ng-dsx* regulatory region increases cheek size

The role of *Ng-dsx* in generating the *N. giraulti* male-specific structures was further tested by taking advantage of an introgression line containing a portion of the regulatory region of *Ng-dsx* isolated into the genetic background of *N. vitripennis* (Figure 3D). The introgression was originally identified as a being important for the larger size of the *Ng* male wing, and was shown to alter *dsx* expression level in wing discs (Loehlin *et al.* 2010). Here, we show that this relatively small introgression (∼40 kb), containing Ng DNA only in the noncoding region upstream of the *dsx* open reading frame [including the promoter and part of the 5' UTR (Loehlin *et al.* 2010)], has a strong effect on the shape of the male head in an otherwise *N. vitripennis* genetic background. For all five measures examined, the introgression line showed highly statistically significant differences to normal *Nv* male values, and trended toward Ng values [*e.g.*, narrower interocular widths, and larger cheeks (Figure 4, *P* < 0.01 for all values, Supplementary Table S3)]. In addition, the introgression line was statistically indistinguishable from normal *Ng* males at MHW/HL and OIO/HL (Figure 4), even though they are genetically Nv except for the introgressed region around *dsx* (Desjardins *et al.* 2013). These findings are consistent with our hypothesis that *dsx* plays a crucial role in generating the *N. giraulti* specific male head shape features.

### Head shape traits have different dominance relations, while head defect alleles are recessive

Due to the obligate haplodiploidy, *Nasonia* males are normally hemizygous, and interactions among alleles can be assessed in the absence of dominance effects. However, understanding the dominance relationships of alleles is helpful in understanding both the function of the genes involved in generating a phenotype, and the molecular nature of interactions that lead to changes or failure in development.

To study the dominance relationships between the two parental genomes while maintaining male-specific traits, we created diploid males using the previously described method of knocking down the maternal *Nv-tra* contribution by pRNAi. In the absence of maternal *Nv-tra*, mated females will produce diploid males (Verhulst *et al.* 2010; Beukeboom *et al.* 2015). Therefore, *Nv-tra* dsRNA injected *Nv* females were mated to *Ng* males, which resulted in diploid, hybrid male offspring (Figure 3E). Since these offspring are F1 hybrids, no genetic recombination or assortment has occurred between the two species' genomes. In addition, no sex-based chromosomal differences in these species, because haplodiploids do not have sex chromosomes. Thus, each hybrid diploid receives an equal contribution of chromosomal genetic material from the parental species.

For all normalized head shape traits measured, the mean values for diploid hybrid males were between those of the parental species (Figure 4, Supplementary Table S3). For two measures, [maximum interocular width (MIO/HL, Figure 4C)] and cheek size (FEP/FE, Figure 4E) the F1 hybrid values were statistically different from both parental species males, indicating incomplete dominance of the alleles governing these traits. For head width across the antennae (AIO/HL, Figure 4D), and maximum head width (MHW/HL, Figure 4A), the F1 hybrid values were not statistically distinguishable from Ng males, which may indicate dominance of Ng alleles governing these traits. Finally, interocular width across the ocelli (OIO/HL, Figure 4C) in diploid hybrid males was statistically indistinguishable from Nv but significantly different from Ng, indicating dominance of the Nv alleles governing this trait.

In contrast, diploid males did not display any of the abnormal phenotypes that occur in haploid hybrids, such as midline clefting and, head asymmetry, indicating that it is not the mere presence of an allele from the other species that causes the developmental defects. Rather, it appears that hybrid head defects involve recessive epistatic interactions among loci from the two species, rather than incompatibilities within individual loci.

### Patterns of hybrid defects among three *Nasonia* species crosses reveal the timing of developmental incompatibilities

**Figure 5:**
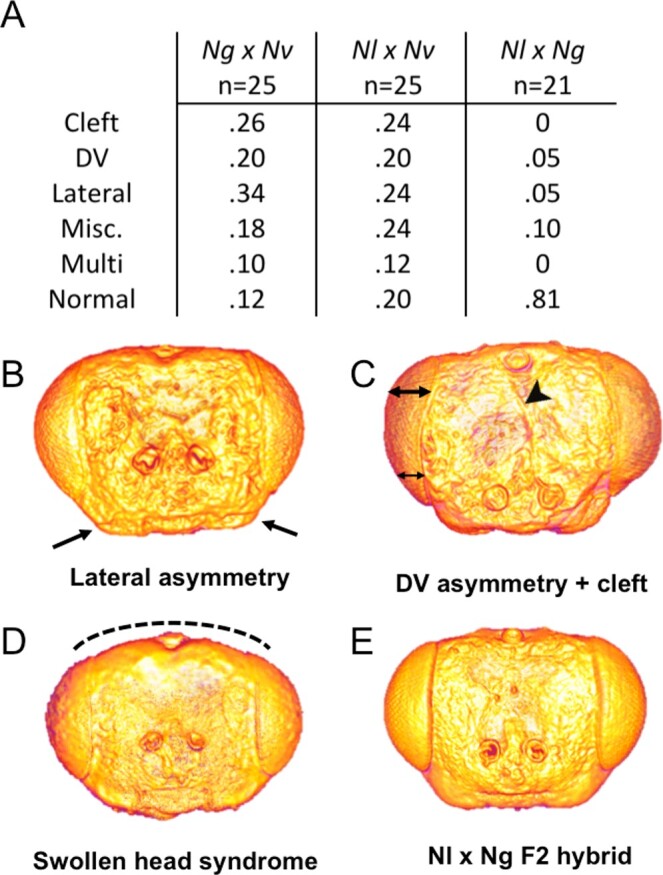
Representative hybrid head shapes from *N. longicornis* crosses. (A) Table containing percentages of hybrid offspring that display each category of facial defect for the three hybrid crosses. The first three categories are facial clefting, dorsoventral asymmetry, and lateral asymmetry. Individuals displaying more than one type of defect are noted under Multi. Miscellaneous defects include swollen head syndrome, bulging eye syndrome, and antennal pits. (B–E) *N. longicornis* x *N. vitripennis* hybrids. (B) Lateral asymmetry, arrows point to differences in cheek size. (C) DV asymmetry and midline cleft, double-ended arrows indicate changes in width of eye field from dorsal to ventral side of the head. Arrowhead points to midline cleft. (D) Swollen head syndrome, the top of the head bulges outward. (E) *N. longicornis* x *N. giraulti* hybrid. Note no obvious aberrations.

**Figure 6:**
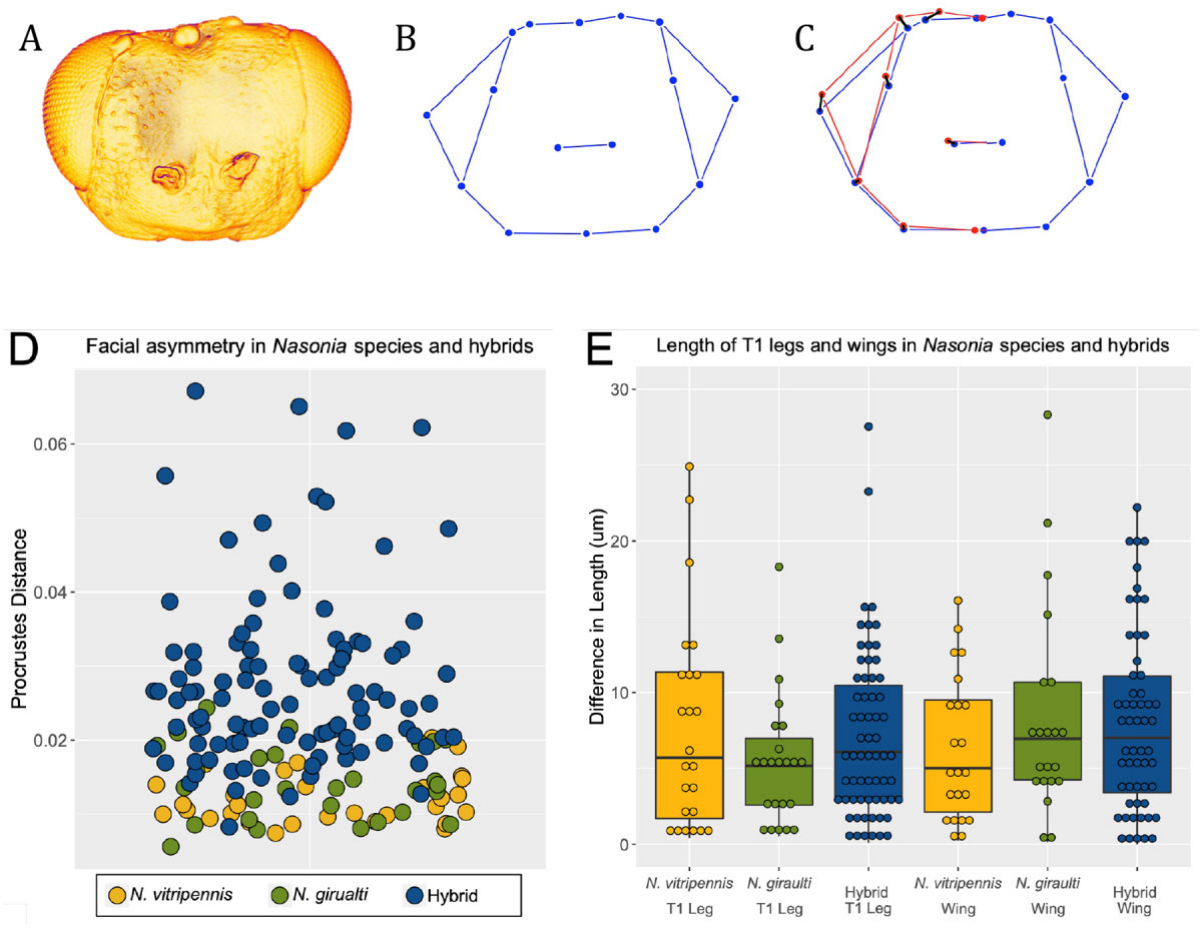
Symmetry analyses. (A) Representative asymmetric hybrid head. (B) Wireframe diagram of head in (A). (C) Right-side landmarks reflected over left side landmarks. Reflection is shown in red. A black line represents distance between corresponding landmarks. Procrustes distance is calculated as the sum of the squares of each distance. (D) Scatter plot in which each dot depicts Procrustes distance for individual wasps. Dark blue dots represent hybrid individuals; yellow, green, and light blue are wild types. *P* < 0.001 between hybrids and wild types. (E) Box Plot graphing differences in length of T1 legs and first set of wings in the same wild type and hybrid wasps as panel (D). ANOVA analysis reveals no significant asymmetry in legs and wings. (*P* = 0.28 among legs and *P* = 0.65 among wings).

F2 hybrid males resulting from Nv-Ng crosses display frequent developmental defects in head morphology (Werren *et al.* 2016, Figure 5A). These defects take several forms, including facial clefting, where a deep furrow forms along the midline of the face (Figure 5C, arrowhead); lateral asymmetry, where structures on either side of the face are displaced and/or of different sized relative to the other side of the face (Figures 5B and 6A); and dorso-ventral asymmetry, where the borders of one or both of the eyes are not parallel with the dorso-ventral axis of the face (Figure 5C). There is also a set of defects that appear at lower frequency (tabulated as “Misc.” in Figure 5A). These include swollen head syndrome, an expansion at the top of the head (Figure 5D); bulging eye syndrome, where the eye field is larger than average causing the facial area to be smaller than average; pitting around the antennal sockets; and presence of a fourth ocellus. Some individuals display more than one type of abnormality, which are noted under “multi” in Figure 5A.

One possible explanation for these hybrid defects is that they are due to the accumulation of genetic substitutions in the Ng and Nv lineages that are buffered in the pure species, but that disrupt molecular/developmental interactions critical for normal head development when brought together in hybrids between Ng and Nv. These would be examples of Dobzhansky-Mueller type hybrid incompatibilities, but resulting in developmental defects rather than sterility or lethality, the usual focus of interspecies genetic incompatibility studies (Figure 2). The evolved head morphological divergence between *Nasonia* species may also contribute to head defects in hybrids (Figure 2).

*N. longicornis* is a sister species of *N. giraulti* relative to *N. vitripennis* (Figure 1). To investigate the timing of head developmental incompatibilities, we further examined F2 hybrid males created with *N. longicornis* (*Nl*)*. Nl* diverged ∼0.4 MYA from *N. giraulti*, while the divergence time between *Nl* and *Nv* is the same as between *Ng* and *Nv* (∼1.4 MYA). Male *Nl* heads are intermediate between Nv and Ng in both normalized narrowness of the face (MIO/HL, Figure 2C), and in relative size of the cheeks (FEP/FE, Figure 2E). Eighty percent of F2 hybrid males produced by Nl-Nv hybrid females exhibit head defects (Figure 5A). This is similar and not significantly different (Chi-Square, *P* > 0.05) from the high rate (88%) of defects observed in Ng-Nv F2 hybrid males (Figure 5A). Thus, although Nl head shape is intermediate between Nv and Ng, the frequency of defects is similar in Nl-Nv and Ng-Nv hybrid F2 males. The rates of the different defect types were also similar, with the Nl-Nv hybrids showing slightly (but not significantly) lower rates of clefting and lateral asymmetry (Figure 5A).

In contrast, head defects are seen in only ∼20% of Ng-Nl hybrid F2 males (Figure 5, A and E). Strikingly, the clefting phenotype was completely absent and both DV and lateral asymmetries only occurred in five percent of Nl-Ng hybrids (compared to ∼24–34% in hybrids from crosses to Nv, Figure 5A). Miscellaneous defects accounted for 10% of Nl-Ng F2 hybrid male heads, accounting for 50% of abnormal phenotypes, while this category accounts for ∼20% of Nv-Nl and Nv-Ng hybrids heads, (Figure 5A) and no individuals of this cross had more than one defect (compared to 10–12% of Nv hybrids, Figure 5A).

A reasonable interpretation of the findings is that that most of the alleles causing developmental defects in the heads of hybrids between Nv and Nl or Ng arose and were fixed prior to the divergence of the Ng and Nl lineages from each other approximately 400,000 years ago (Campbell *et al*. 1993; Martinson *et al.* 2017). The defects seen in Nl-Ng hybrids (at low frequency) may be due to new alleles that have arisen in one or both lineages, or may reflect independent sorting of polymorphisms present in the ancestral population that gave rise to them.

### Developmental asymmetry is restricted to heads of hybrid males

The most common of the abnormal hybrid head phenotypes is morphological asymmetry (Figure 5A). We sought to determine whether the asymmetries were caused by a general developmental instability in the hybrids, as is seen in some systems (Alibert and Auffray 2003; Leamy and Klingenberg 2005), or if the phenotype had its basis in genetic mechanisms operating specifically in the head. To determine this, we developed an approach to quantify asymmetry among head capsules as well as difference in length at two other body parts: legs and wings (Figure 6E). Symmetry between left and right sides of heads was quantified by overlaying landmarks from the left to their corresponding landmarks on the right (*i.e.*, the wireframe is folded along the centerline) and a Procrustes distance analyses is performed by calculating **Σ**[(distance between corresponding landmarks)^2^]. A Procrustes distance analysis (Figure 6, A–C) done on 105 *Nv × Ng* hybrid heads found that a hybrid head has only 93% correlation on average between its left and right sides (Figure 6D). On the other hand, wild type heads measured from both males and females of *N. vitripennis* and *N. giraulti* revealed a 99.5% correlation between left and right sides of the head. The differences in symmetry are statistically significant (*P* < 0.001, by *t*-test of individual Procrustes distances). However, we found no significant difference in the length between the left and right T1 legs, nor between the forewings in the same set of F2 hybrid wasps, as compared to either parental species (ANOVA *P* = 0.28 among legs and *P* = 0.65 among wings, Figure 6E, Supplementary Table S4).

The next most common developmental defect in F2 hybrid males is the facial clefting phenotype (Figure 5, A and C). As described above, this phenotype is characterized by a deep fissure or in-folding of the epidermis along the vertical midline of the face. No such defects appear on either the thorax or abdomen of these wasps (not shown), and these hybrids appear to develop normally posterior to the head.

Given the restriction of these two major developmental incompatibilities to the head, we propose that generalized developmental instability is not a likely explanation for cranial asymmetry or midline clefting. Rather, these phenotypes appear to stem from a phenomenon specific to the head patterning and development system, likely arising due to divergence in development and male head shape between the species.

### Genetics of the abnormal clefting phenotype

As shown above [and previously (Li *et al.* 2005; Desjardins *et al.* 2010; Loehlin *et al.* 2010; Loehlin and Werren 2012; Hoedjes *et al.* 2014)], introgression of genomic regions from one species' background into another is a powerful method to analyze the genetic basis of evolutionary traits in *Nasonia*. Previous QTL analyses for head clefting showed a complex web of genetic interaction among regions on Chrs 2, 4, and 5 (Werren *et al.* 2016). Briefly, clefting occurs at a frequency of ∼25% when either or both the regions on Chr 2 and Chr 4 have the *N. giraulti* genotype AND the region on Chr 5 has the *N. vitripennis* genotype. If Chr 5 has the *N. giraulti* genotype, clefting is completely suppressed, unless both the Chr 2 and Chr 4 region derives from *N. vitripennis*. Clefting also occurs at about 25% of the time when all three regions derive from *N. vitripennis*, indicating that at least one more locus is involved, or that there is an effect of the general hybrid background on the threshold for clefting.

To simplify analysis of this trait, we examined existing introgression lines with segments of *Ng* DNA introgressed in a *Nv* background. One line, derived from a larger introgression spanning the centromere of Chr 2 consistently showed facial clefting (see *Materials and Methods*, Figure 3F). Significantly, the females homozygous for this introgression also display the cleft phenotype, unlike F1 hybrid females that never show abnormalities. This indicates that interactions leading to the epistatic phenotype are recessive but not sex specific, since the introgression lines are homozygous and the trait is not seen in the F1 females. The result is also consistent with the F2 clefting QTL analysis, which predicts that the *Ng* allele in Chr 2 will induce clefting when combined with the *Nv* alleles at the locus on Chrs 4 or 5 (Werren *et al.* 2016), because the introgression line is fixed for Nv genes in these two regions. This result also indicates that the clefting trait is not directly related to the sex-specific morphological divergence between the species, and is rather a general defect in head development. Finally, this introgression shows that, at least for the locus on Chrs 2, the clefting trait is fully penetrant when the Ng locus is backcrossed into an Nv background. This will simplify identification of the causative allele from Ng and fine-scale mapping and positional cloning of suppressing/interacting alleles at other loci (*e.g.*, on Chr 5).

## Discussion

Studies have found that the key determinant in primary sex determination in metazoans, *doublese*x, plays an important role in evolutionary changes in sexually dimorphic traits within and between species (Kopp 2012), such as horn size in dung beetles (Rohner *et al.* 2021), mimicry in butterfly wings (Kunte *et al.* 2014), wing size (Loehlin *et al.* 2010), and antennal pigmentation (Wang *et al.* 2020) in *Nasonia*. Here, we show that *dsx* also has an important role in sexual differences in male head shape between closely related *Nasonia* species. First, knockdown of *Ng-dsx* decreases male-specific differences in head morphology in Ng. Second, introgression of a cis-regulatory element from Ng into the Nv background induces partial transformation to an Ng head shape. Since neither the *Ng-dsx* RNAi nor the *Ng-dsx* genomic introgression led to complete transformation [to Ng female, or Ng male, respectively (Figures 3 and 4)], it is clear that other factors are involved in mediating species-specific features of male head shape. It is likely that multiple loci contribute significantly to the head shape differences, some of which are under the influence of *dsx* and others that are not. The same pattern is found for male wing size and shape network differences between these two species (Gadau *et al.* 2002; Loehlin *et al.* 2010; Loehlin and Werren 2012). Indeed, a complex genetic bases for all of the differing male head shape and size features were predicted in our previous QTL analysis (Werren *et al.* 2016).

We also investigated the genetic basis of head abnormalities found in hybrids. While head morphology is strongly influenced by sex, the most frequent developmental defect in F2 hybrid males (clefting) is not, since our clefting introgression line containing a Ng locus in a Nv genetic background (Figure 3F) shows that the phenotype occurs in homozygous females with complete penetrance, as well as in haploid males. The effect of this locus on clefting depends upon interacting Nv alleles. A future goal is to uncover the set of interacting loci from Ng and Nl that can result in head clefting originally detected in a QTL analysis of F2 hybrid males (Werren *et al.* 2016), and this promises to be a good system for unraveling complex genetic interactions underlying morphological development, and particularly for abnormalities in development.

There are likely to be different genetic interactions at play for the suite of developmental defects observed in F2 hybrid males. For example, asymmetric phenotypes in hybrid F2 males are examples of fluctuating asymmetries (FA). FA is generally considered a proxy measurement for developmental instability (Valen 1962; Dongen 2006). Developmental instability can result from any number of genetic [including interspecies hybridization) (Leamy and Klingenberg 2005)], epigenetic or environmental factors, and the ability of an organism to buffer extrinsic insults to produce symmetric form has also been proposed to be a proxy of fitness (Clarke 1995). This idea continues to be controversial (Lens *et al.* 2002; Leamy and Klingenberg 2005), as it has been observed that not all traits have the same susceptibility to FA (Valen 1962; Aparicio and Bonal 2002). It has been proposed that complex structures with critical functions and low tolerance for deviations in shape may be subject to stronger selection to preserve symmetry (Palmer and Strobeck 1986; Aparicio and Bonal 2002). The head is an obvious case for this. Based on these ideas, we propose the head asymmetries we observe in F2 hybrid males are the result of disrupting allele interactions that have been strongly, but divergently, selected in the Nv and Ng/Nl lineages to maintain facial symmetry.

The feasibility of dissecting gene interactions governing complex head defects using introgression and recombination mapping has already been shown with our work with the clefting trait, so *Nasonia* is also well positioned to make a valuable contribution to understanding the genetic basis of developmental buffering asymmetry.

In crosses between closely related flies *Drosophila simulans and D. mauritiana*, which have divergent head shapes, seemingly coordinated changes in size of the eye field and facial cuticle were found to be due to separable genomic loci (Arif *et al*. 2013). No complex gene interactions or developmental defects (such as clefting or asymmetry) were reported. This may be due to the shorter divergence time between the *Drosophila* species [∼250,000 years (Garrigan *et al*. 2012)] than between *N. vitripennis and N. giraulti/N. longicornis* (∼1.4 million years). Future analyses may reveal whether differences between *N. longicornis and N. giraulti* have more simple genetic bases, like those observed between *D. simulans and D. mauritiana*, or whether complex epistasis is already a factor after a relatively short time of divergence (∼400,000 years between Nl and Ng).

The genetic features of the *Nasonia* system provide a realistic prospect for determining the genes underlying differences in shape and developmental incompatibilities (and their interactions). QTL analysis is valuable as a starting point for fine-scale mapping of interacting loci that are the genetic basis for observed disrupted phenotypes. Putative causal regions can be isolated in the other species' genetic background by introgression for further analysis, followed by positional cloning, as already accomplished in *Nasonia* for different phenotypes (Niehuis *et al.* 2013; Funkhouser-Jones *et al.* 2018). For head shape genetics, our identification of the completely penetrant major hybrid clefting locus is a major step toward identifying causal genes in head abnormalities. Fine-scale mapping and expression studies will help to identify the causal locus, and the region can be used as a tool to “capture” other interacting loci that rescue the phenotype, by introgression from Ng. Use of the diploid male method can reveal the level of dominance and penetrance of these loci for cleft production. Thus, there is a reasonable program for unraveling the complex genetic basis of this abnormal developmental phenotype.

Introgression is a very useful method to understand quantitative traits and gene interactions, whereby a section of one genome is isolated in the background of another through a series of backcrosses, and its localized effects examined. Introgression lines are also powerful starting points for fine scale mapping and positional cloning of causative alleles. The introgression of the clefting locus on Chr 2 is a good example of the power of the introgression approach. Given the complexity of the interactions that govern the appearance of the cleft in F2 hybrid males, it was somewhat surprising that the introgression of the *N. giraulti* Chr 2 locus led to a completely penetrant phenotype in both males and females, behaving basically as a Mendelian recessive allele. Thus, it appears that while the genetic architecture preventing clefting in the pure species is complex, each individual allele may have a relatively simple and robust role, rather than each locus having an unpredictable magnitude of effect on the phenotype.

Future analyses will focus on determining whether the other participating alleles predicted by the QTL analyses (Werren *et al.* 2016) also have strong effects in a foreign background, or if there is a mixture of completely and incompletely penetrant negative interactions. In particular, based on the QTL analysis, (Werren *et al.* 2016), a region on Chr 5 interacts with the region from Chr 2. We therefore expect an introgression of the Chr 5 region to completely suppress clefting by the locus in the Chr 2 introgression, since clefting occurred 0% of the time when these two alleles were present together in F2 males used for the QTL analysis. The expected phenotype of this Chr 5 region is less clear, since overall clefting occurred 25% of the time when regions on both Chr 2 and Chr 4 had the *N. vitripennis* genotype (Werren *et al.* 2016). This indicates either that there are other loci that suppress clefting induced by the *N. giraulti* Chr 5 allele, or that this allele does not promote clefting in a fully penetrant way. The tools available in *Nasonia* will allow us to resolve this question one way or the other.

## Conclusion

The genetic tools available in *Nasonia* and availability of haploid males, combined with the complex genetic architectures of head shape and developmental defects, makes *Nasonia* a promising system for investigating the microevolution of complex genetic traits in closely related species.

## Data availability

Strains are available upon request. Supplementary Figure S1 shows how heads were measured. Supplementary Table S1 provides the raw measurements of the parental species heads. Supplementary Table S2 provides a side-by-side comparison of the measurements of parental and experimental heads. Supplementary Table S3 provides the measurements of the experimental strain heads. Supplementary Table S4 gives the measurements of the wings and legs of parental species and hybrid wasps. The authors affirm that all data necessary for confirming the conclusions of the article are present within the article, figures, and tables.

Supplementary material is available at *G3* online.

## Funding

Support for J.A.L. was provided by National Institutes of Health grants R01GM129153 and R03HD087476. Support for J.H.W. comes from National Institutes of Health grant GM70026, National Sciene Foundation Grants IOS-1456233, NSF 1950078, and the Nathaniel and Helen Wisch Chair in Biology.

## Conflicts of interest

The authors declare that there is no conflict of interest.

## Supplementary Material

jkab313_Supplementary_Figure_S1

jkab313_Supplementary_Table_S1

jkab313_Supplementary_Table_S2

jkab313_Supplementary_Table_S3

jkab313_Supplementary_Table_S4
