# Supplementary material for: Genetic, morphometric, and molecular analyses of interspecies differences in head shape and hybrid developmental defects in the wasp genus Nasonia
Source: G3 (Bethesda). 2021 Sep 2;11(12):jkab313. doi: 10.1093/g3journal/jkab313 (PMC8664464; doi:10.1093/g3journal/jkab313)
Supplement: jkab313_Supplementary_Figure_S1 [file jkab313_supplementary_figure_s1.pdf]

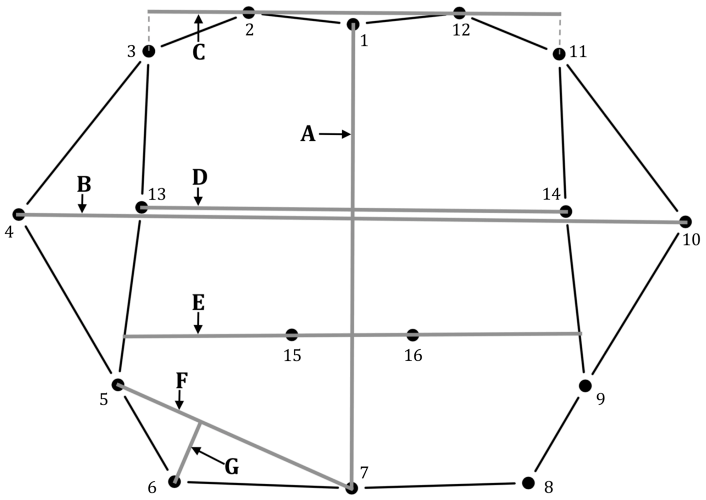

Figure S1. How heads were measured. Each numbered black dot represents a landmark. Landmarks 2 and 12 are at lateral ocelli; 3, 5, 9, 11, 13 and 14 mark where optical tissue meets head capsule; 4 and 10 represent maximum arc of each eye; 6 and 8 are at maximum arc of cheeks; and landmarks 15 and 16 are antennal sockets. A) HL- head length down center of face, from landmark 1 to landmark 7. B) MHW- maximum head width, from landmark 4 to landmark 10. C) OIO- interocular distance through ocelli directly above landmarks 3 and 11. D) MIO- maximum interocular distance, landmark 13 to landmark 14. E) AIO- interocular distance across antennal sockets, from where eye tissue meets head capsule across landmarks 15 and 16. F) FE- distance from bottom of eye to center of mandible, landmark 5 to landmark 7. G) FEP- farthest point on cheek, landmark 6, perpendicular to line FE. Measurements are presented as ratios to normalize natural difference in overall size of the individual. B-E are divided by A, and G divided by F.
