# Supplementary material for: Genetic, morphometric, and molecular analyses of interspecies differences in head shape and hybrid developmental defects in the wasp genus Nasonia
Source: G3 (Bethesda). 2021 Sep 2;11(12):jkab313. doi: 10.1093/g3journal/jkab313 (PMC8664464; doi:10.1093/g3journal/jkab313)
Supplement: jkab313_Supplementary_Table_S1 [file jkab313_supplementary_table_s1.docx]

**Table S1. Measurement ratios of each of the parent species and their significance from each other.**

|  | Males | | | Females | | | Significance by Utest | | |
| --- | --- | --- | --- | --- | --- | --- | --- | --- | --- |
|  | *N. vitripennis* | *N. giraulti* | *N. longicornis* | *N. vitripennis* | *N. giraulti* | *N. longicornis* | males | females | sex w/in species |
|  | n=18 | n=16 | n=14 | n=19 | n=20 | n=12 | v-g/g-l/v-l | v-g/g-l/v-l | v/g/l |
| MHW/HL | 1.63 ±0.03 | 1.38 ±0.05 | 1.34 ±0.09 | 1.40 ±0.05 | 1.35 ±0.05 | 1.29 ±0.06 | ***/ - /*** | **/*/*** | ***/ - / - |
|  |  |  |  |  |  |  |  |  |  |
| OIO/HL | 0.91 ±0.04 | 0.83 ±0.03 | 0.84 ±0.09 | 0.91 ±0.05 | 0.90 ±0.04 | 0.87 ±0.07 | ***/ - /* | - / - / - | - /***/ - |
|  |  |  |  |  |  |  |  |  |  |
| MIO/HL | 1.22 ±0.03 | 0.89 ±0.03 | 1.00 ±0.08 | 1.09 ±0.04 | 0.99 ±0.03 | 1.03 ±0.07 | ***/***/*** | ***/ - / - | ***/***/ - |
|  |  |  |  |  |  |  |  |  |  |
| AIO/HL | 1.00 ±0.04 | 0.89 ±0.03 | 0.91 ±0.06 | 0.99 ±0.04 | 0.98 ±0.03 | 0.97 ±0.03 | ***/ - /*** | - / - / - | - /***/** |
|  |  |  |  |  |  |  |  |  |  |
| FEP/FE | 0.12 ±0.01 | 0.23 ± 0.03 | 0.19 ±0.03 | 0.12 ±0.02 | 0.14 ±0.02 | 0.15 ±0.02 | ***/**/*** | ***/ - /** | - /***/** |

**Values represent average per group ± standard deviation. Single asterisks indicate P<0.05, double asterisks indicate P<0.01, and triple asterisks indicate P<0.001.**
