# Supplementary material for: Genetic, morphometric, and molecular analyses of interspecies differences in head shape and hybrid developmental defects in the wasp genus Nasonia
Source: G3 (Bethesda). 2021 Sep 2;11(12):jkab313. doi: 10.1093/g3journal/jkab313 (PMC8664464; doi:10.1093/g3journal/jkab313)
Supplement: jkab313_Supplementary_Table_S2 [file jkab313_supplementary_table_s2.docx]

|  | wildtype *N. giraulti* | | experimental | significance | |
| --- | --- | --- | --- | --- | --- |
|  | female | male | *N.g. dsx* RNAi | exp-fem | exp-male |
|  | n=20 | n=16 | n=19 |  |  |
| MHW/HL | 1.35 ±0.05 | 1.38 ±0.05 | 1.40 ±0.08 | ** | - |
|  |  |  |  |  |  |
| OIO/HL | 0.90 ±0.04 | 0.83 ±0.03 | 0.88 ±0.05 | - | * |
|  |  |  |  |  |  |
| MIO/HL | 0.99 ±0.03 | 0.89 ±0.03 | 1.03 ±0.06 | ** | *** |
|  |  |  |  |  |  |
| AIO/HL | 0.98 ±0.03 | 0.89 ±0.03 | 0.97 ±0.04 | - | *** |
|  |  |  |  |  |  |
| FEP/FE | 0.14 ±0.02 | 0.23 ± 0.03 | 0.19 ±0.02 | *** | ** |

**Table S2. Measurement ratios of *Ng dsx* RNAi experimental strain as compared to both male and female wildtype *N. giraulti*.**

Values represent average per group ± standard deviation. Single asterisks indicate P<0.05, double asterisks indicate P<0.01, and triple asterisks indicate P<0.001.
