# Supplementary material for: Genetic, morphometric, and molecular analyses of interspecies differences in head shape and hybrid developmental defects in the wasp genus Nasonia
Source: G3 (Bethesda). 2021 Sep 2;11(12):jkab313. doi: 10.1093/g3journal/jkab313 (PMC8664464; doi:10.1093/g3journal/jkab313)
Supplement: jkab313_Supplementary_Table_S3 [file jkab313_supplementary_table_s3.docx]

**Table S3. Measurement ratios of experimental strains.**

|  | wild type males | | Experimental males | | | | Significance by Utest | |
| --- | --- | --- | --- | --- | --- | --- | --- | --- |
|  | *N. vitripennis* | *N. giraulti* | Diploid hybrid | *N.g. dsx* RNAi | Chromosome 4 introgression | Chromosome 2 introgression | exp-*Nv* | exp-*Ng* |
|  | n=18 | n=16 | n=14 | n=19 | n=20 | n=12 | diploid/RNAi/chr4/chr2 | diploid/RNAi/chr4/chr2 |
| MHW/HL | 1.63 ±0.03 | 1.38 ±0.05 | 1.40 ±0.04 | 1.40 ±0.08 | 1.42 ±0.05 | 1.52 ±0.09 | ***/***/***/** | - / - / - /** |
|  |  |  |  |  |  |  |  |  |
| OIO/HL | 0.91 ±0.04 | 0.83 ±0.03 | 0.91 ±0.02 | 0.88 ±0.05 | 0.83 ±0.05 | 0.88 ±0.07 | - / - /***/ - | ***/*/ - /* |
|  |  |  |  |  |  |  |  |  |
| MIO/HL | 1.22 ±0.03 | 0.89 ±0.03 | 1.00 ±0.05 | 1.03 ±0.06 | 0.97 ±0.03 | 1.14 ±0.09 | ***/***/***/* | ***/***/***/*** |
|  |  |  |  |  |  |  |  |  |
| AIO/HL | 1.00 ±0.04 | 0.89 ±0.03 | 0.91 ±0.05 | 0.97 ±0.04 | 0.94 ±0.02 | 0.92 ±0.06 | ***/ - /***/** | - /***/***/ - |
|  |  |  |  |  |  |  |  |  |
| FEP/FE | 0.12 ±0.01 | 0.23 ± 0.03 | 0.17 ±0.02 | 0.19 ±0.02 | 0.16 ± 0.01 | 0.14 ±0.02 | ***/***/***/ - | **/**/***/*** |

First two rows are wild type males of *N. vitripennis* and *N. giraulti*. Values represent average per group ± standard deviation. Single asterisks indicate P<0.05, double asterisks indicate P<0.01, and triple asterisks indicate P<0.001.
