# Supplementary material for: Genetic, morphometric, and molecular analyses of interspecies differences in head shape and hybrid developmental defects in the wasp genus Nasonia
Source: G3 (Bethesda). 2021 Sep 2;11(12):jkab313. doi: 10.1093/g3journal/jkab313 (PMC8664464; doi:10.1093/g3journal/jkab313)
Supplement: jkab313_Supplementary_Table_S4 [file jkab313_supplementary_table_s4.docx]

|  | average leg difference (um) | average wing difference (um) |
| --- | --- | --- |
| *N. vitripennis* | 7.71 | 6.32 |
| *N. giraulti* | 5.53 | 8.40 |
| hybrids | 8.53 | 7.97 |
|  | P=0.28 | P=0.65 |

**Table S4. Average difference in length of T1 legs and first set of wings in wild type male wasps and hybrid males**
